# The global pandemics are getting more frequent and severe

**DOI:** 10.34172/jrhs.2021.40

**Published:** 2021-01-18

**Authors:** Jalal Poorolajal

**Affiliations:** ^1^Research Center for Health Sciences, School of Public Health, Hamadan University of Medical Sciences, Hamadan, Iran; ^2^Department of Epidemiology, School of Public Health, Hamadan University of Medical Sciences, Hamadan, Iran

**Figure 1:**
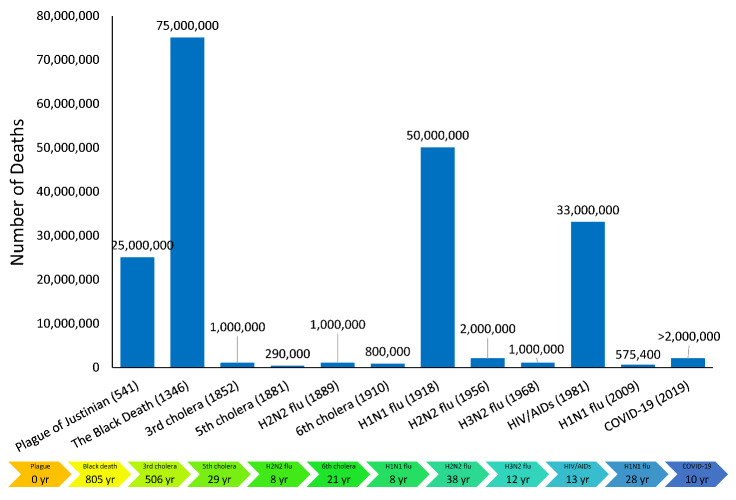
Number of deaths due to 12 deadliest pandemics of the world (excluding SARA in 2002 and 7^th^ Cholera pandemics in 1961 due to less than 10,000 deaths), the time intervals between pandemics are shown below the figure

Evidence shows that deadly pandemics have been happening more often in recent decades^1,2^. As shown in Figure 1, 5 out of 12 deadly pandemics occurred in the 20^th^ century and the other 2 in the 21^st^ century. The last four pandemics occurred almost every 15 years on average. It seems that the occurrence of pandemics getting more frequent over time. It warns us that deadly pandemics are likely to keep happening, spread faster, and kill more people than Coronavirus Disease 2019 (COVID-19) soon if we do not take appropriate preemptive global action to protect natural environments. Such pandemics can be a dangerous threat to the world economy and long-lasting harm to social and cultural life.

The Centers for Disease Control and Prevention (CDC) has estimated that six out of every 10 known infectious diseases that affect people can be spread from animals and three out of four new or emerging infectious diseases come from animals to humans^3^.

A report from the Intergovernmental Science-Policy Platform on Biodiversity and Ecosystem Services indicated that almost 70% of emerging diseases, such as Ebola, Zika, Nipah encephalitis, and almost all known world pandemics, such as influenza, HIV/AIDS, and COVID-19 are zoonoses that are caused by microorganisms of animal origin (e.g. bats, rodents, primates) and some birds (e.g. water birds), as well as livestock (e.g. pigs, camels, poultry)^4^.

It is expected that COVID-19 probably originated from bats and began spreading among people at a market in Wuhan, China^5^. Experts believe that the exploitation of the environment due to land-use changes, deforestation, agriculture expansion, wildlife trade, and consumption, as well as urbanization, can disrupt natural interactions among wildlife and their microbes; moreover, it increases contacts among wildlife, livestock, and human which may lead to new infectious diseases and pandemics in the world^4^.

The current approach for dealing with new emerging diseases and pandemics is to wait until they happen and then try to restrain them before they spread. The way we dealt with the COVID-19 pandemic and its strange geographical distribution around the world^6^ is a clear example of such a problematic approach. A majority of efforts aim to contain pandemics after they happen rather than trying to prevent them. Worldwide efforts have to be taken to stop environmental and climate changes that can lead microorganisms to jump from wild animals to humans; otherwise, more frequent and severe pandemics will emerge in the future.
